# Mice and rats achieve similar levels of performance in an adaptive decision-making task

**DOI:** 10.3389/fnsys.2014.00173

**Published:** 2014-09-18

**Authors:** Santiago Jaramillo, Anthony M. Zador

**Affiliations:** ^1^Cold Spring Harbor LaboratoryCold Spring Harbor, NY, USA; ^2^Institute of Neuroscience and Department of Biology, University of OregonEugene, OR, USA

**Keywords:** auditory, categorization, psychometric, context, flexibility, task switching, cognition

## Abstract

Two opposing constraints exist when choosing a model organism for studying the neural basis of adaptive decision-making: (1) experimental access and (2) behavioral complexity. Available molecular and genetic approaches for studying neural circuits in the mouse fulfill the first requirement. In contrast, it is still under debate if mice can perform cognitive tasks of sufficient complexity. Here we compare learning and performance of mice and rats, the preferred behavioral rodent model, during an acoustic flexible categorization two-alternative choice task. The task required animals to switch between two categorization definitions several times within a behavioral session. We found that both species achieved similarly high performance levels. On average, rats learned the task faster than mice, although some mice were as fast as the average rat. No major differences in subjective categorization boundaries or the speed of adaptation between the two species were found. Our results demonstrate that mice are an appropriate model for the study of the neural mechanisms underlying adaptive decision-making, and suggest they might be suitable for other cognitive tasks as well.

## 1. Introduction

A central challenge of modern neuroscience is to understand the neural mechanisms underlying behavior. Historically, macaque monkeys have served as the preeminent non-human experimental preparation for studying complex cognitive behaviors such as attention and decision making (Desimone and Duncan, 1995; Parker and Newsome, 1998; Maunsell and Treue, 2006; Gold and Shadlen, 2007). Data derived from this preparation form the foundation for most modern theories of neural computation. Progress in primate systems neuroscience has been propelled largely by sophisticated conceptual tools for data analysis and computational modeling. However, because of the great value of individual animals, primate research is in many ways technologically conservative. These limitations have slowed the pace of progress in systems neuroscience.

Rats have also been an important experimental model for studying cognition (Tolman, 1948; Morris, 1984; Dudchenko, 2004; Izquierdo and Belcher, 2012). Over the last decade, we and others have developed sensory psychophysics and decision-making paradigms for rats explicitly modeled after those used in primates (Uchida and Mainen, 2003; Kepecs et al., 2008; Otazu et al., 2009; Erlich et al., 2011; Jaramillo and Zador, 2011; Raposo et al., 2012; Znamenskiy and Zador, 2013; Jaramillo et al., 2014). Because of their low cost—at least two orders of magnitude lower than monkeys—it is possible to conduct physiological and behavioral assays in rodents in parallel, much more efficiently and rapidly than primates. Moreover, it is straightforward to exploit many of the recent powerful optogenetic tools (Zhang et al., 2010) in rats. However, mice offer further advantages over rats, stemming mainly from the availability of hundreds of transgenic lines that enable precise targeting of gene expression to defined neuronal populations (Madisen et al., 2010; Taniguchi et al., 2011; Madisen et al., 2012; Gerfen et al., 2013). It is therefore crucial to evaluate whether mice can achieve the high levels of performance on cognitive tasks that has previously been demonstrated in rats.

Here we compare the speed of learning and the performance levels achieved by mice and rats in a decision-making task that tested the animals' perceptual abilities as well as their adaptability (Jaramillo et al., 2014). The task required animals to categorize acoustic stimuli as high- or low-frequency sounds, and to adapt quickly after changes in the category boundary. We found that mice achieved high performance levels in this task similar to that of rats. The distribution of required training sessions for the two species largely overlapped, although on average mice required longer training. Our results also indicate that once animals learn the task, mice and rats adapt equally fast after a change in categorization contingency.

We conclude that this flexible categorization paradigm provides an appropriate model for studying the neural mechanisms underlying adaptive decision-making in the mouse. Moreover, this report serves as a guide for researchers wanting to implement similar complex behaviors in rodents.

## 2. Materials and methods

### 2.1. Animal subjects

Animal procedures were approved by the Cold Spring Harbor Laboratory Animal Care and Use Committee and carried out in accordance with National Institutes of Health standards. Ten adult male Long Evans rats (Taconic Farms) and ten adult male C57Bl/6J mice (The Jackson Laboratory, stock #000664) were used in this study (Table 1). Data from an additional set of 5 adult C57Bl/6J mice from a different study are also presented (**Figure 7**). Animals had free access to food, but water was restricted. Free water was provided on days with no experimental sessions. The weight of animals was monitored at least three times a week, and supplemental water was given if an individual had a weight below 80% of its baseline.

**Table 1:** **Animals in the study**.

	**Rats**	**Mice**
Strain	Long evans	C57Bl/6J
Age	10 weeks	10 weeks
Weight	285 ± 29 g	26.1 ± 1.3 g
Reward/trial	24 μl	2–3 μl

### 2.2. Behavioral apparatus

**Figure 1:**
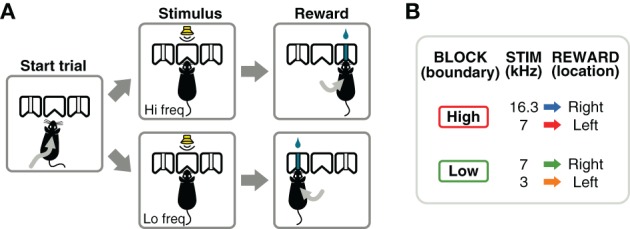
**Flexible sound-categorization task**. **(A)** Subjects initiated each trial by poking their nose into the center port of a three-port chamber. A narrow-band sound was presented for 100 ms indicating the location of reward: left for low-frequency sounds, right for high-frequency sounds. **(B)** Animals were trained using 3 sound frequencies, one of which (7 kHz) changed its meaning several times in a session. After the initial training stages, a behavioral session consisted of alternating blocks of 300 valid trials: in one block type, the category boundary was set to a frequency above 7 kHz; in the other block type the boundary was set to a frequency below 7 kHz.

Experiments were conducted inside single-walled sound-booths with inner dimensions 28 × 26 × 32 inches (W × D × H), custom designed by Industrial Acoustics Company (Bronx, New York). Each booth contained a three-port chamber (Figure 1A) similar to those used by Uchida and Mainen (2003); Otazu et al. (2009); Jaramillo and Zador (2011). For this study, the center port was used for trial initiation and fixation, and the side ports were used for behavioral choices and reward delivery. Nose pokes were detected by sensing the interruption of an infrared beam across each port. Water rewards were delivered via stainless steel tubing (14 gauge for rats, 18 gauge for mice) located in the center of each side port. The amount of water delivered was controlled by valves calibrated at least once a week. Calibration was achieved by estimating the time of valve opening necessary to deliver the desired volume per trial, by running 200 deliveries and calculating water volume by weight. The fluid pressure was not adjusted, but the flow rate changed less than 10% from the beginning to the end of a session. The volume of water reward was scaled according to the normal consumption for each species (Table 1). The behavioral system was controlled by in-house software developed in Matlab (Mathworks, Natick, MA), based on the BControl framework (http://brodylab.princeton.edu/bcontrol).

Sounds were delivered through generic electromagnetic dynamic speakers (HP 5187-2105, Harman Kardon) located on each side of the chamber, and calibrated using a free-field microphone (Type 4939, Brüel and Kjær) to produce 70 dB-SPL in the range of 1–40 kHz at the position of the subject. Measurements were performed with the booth closed and the microphone positioned at the location and orientation of the subject's left or right each when calibrating the left or right speaker respectively. The microphone was connected to a preamplifier (Type 2670, Brüel and Kjær), and signals were digitized with a National Instruments acquisition card (NI 9201) at 500,000 samples per second for analysis with custom software developed in Matlab (Mathworks). A Brüel and Kjær calibrator (Type 4231) that produces 1 kHz at 94 dB-SPL was used to verify the correct output level of the microphone/preamplifier. Waveforms were created in software at a sampling rate of 200,000 samples per second and delivered to speakers through a Lynx L22 sound card (Lynx Studio Technology). We applied rise and fall linear envelopes of 2 ms to all sounds.

### 2.3. Flexible categorization task

After a series of training stages described in the next section, animals were required to perform a frequency discrimination task in which the boundary that separated high- from low-frequency sounds varied within a session. Animals initiated each trial by poking their nose into the center port of a three-port chamber (Figure 1A). After a silent delay of random duration (250–350 ms, uniformly distributed), a narrow-band sound was presented for 100 ms. The silent delay is useful during electrophysiological experiments as a baseline (fixation) period before stimulus presentation. Animals were required to stay in the center port until the end of the sound, and choose one of the two side ports for reward according to the frequency of the sound (low-frequency: left port; high-frequency: right port). If animals withdrew before the end of the stimulus, the trial was aborted and denoted as invalid. Only valid trials were used in calculations of the fraction of correct trials. Left and right trials were fully randomized. *Post-hoc* analysis of the trials presented in each session verified that a strategy of always choosing one side, would yield performances below 60% on 98% of the sessions. Similarly, a strategy of alternating sides would yield performances below 60% on 99.9% of the sessions.

The acoustic stimuli were chords composed of 16 simultaneous pure tones logarithmically spaced in the range *f*/1.2 to *f* × 1.2, for a given center frequency *f*. The intensity of the sounds was variable in the range 50–70 dB-SPL to discourage the use of loudness to solve the task; the perceived loudness of a sound depends on frequency even when the same sound-pressure level reaches the ear. Although rats and mice have different absolute thresholds for the frequencies used here: 0–20 dB-SPL for rats (Heffner et al., 1994) and 10–40 dB-SPL for mice (Koay et al., 2002), all sounds were presented above threshold for either species. Animals were trained to discriminate between sounds centered at 3 and 7 kHz in one contingency, and 7 and 16.3 kHz in another contingency (Figure 1B). These frequencies were chosen to obtain equal (logarithmic) separation between the two frequencies on each contingency. A single session (1 h) consisted of several blocks of 300 valid trials. The categorization contingency changed from one block to the next without any additional cue indicating the change (besides the sound-action-reward relations). The initial contingency for each session was alternated from one day to the next.

### 2.4. Training stages

Animals passed through 7 stages of training, advancing from one stage to the next once they achieved a performance criterion specific to each transition (Table 2). Session at all stages were 1 h long. The first two stages familiarized animals with the behavioral setup for operant conditioning: In the initial stage, 1-DS (Direct Sides), water reward was delivered from one of the two reward ports when the animal poked either on the center port or on the appropriate port for that trial (randomized from trial to trial). A sound associated with the reward port was presented (3 kHz for left, 16.3 kHz for right). Animals advanced to the next stage after the first session with more than 200 rewards. Stage 2-DC (Direct Center) was similar to 1-DS, but reward was only delivered after poking on the center port. Animals advanced to the next stage after the first session with more than 200 rewards.

**Table 2:** **Training stages**.

**Stage**	**Name**	**Goal**	**Water delivery**	**Criterion to advance**
1-DS	Direct sides	Get animals to poke and collect water	After center or side poke	One session with 200 rewards
2-DC	Direct center	Trial initiation in center poke	After center poke	One session with 200 rewards
3-RS	Require side	Require response shortly after trial initiation	After trial initiation and associated side poke	One session with 200 trials
4-ID	Increase delay	Require animals to stay in center port longer	After trial initiation and associated side poke	One session with 200 trials, or 3 consecutive sessions with more than 100 trials
5-RC	Require correct	Full two-alternative choice sound discrimination	Only after correct side poke	Two consecutive sessions with 80% correct for each stimulus.
6-SB	Switch in blocks	Full flexible categorization task, switching every 300 trials	Only after correct side poke (switches for one sound)	One session with 70% correct for the reversing stimulus
7-OK	Ready	Ready for psychometric measurements	Only after correct side poke (category boundary switches)	

The next two stages familiarized animals with the task structure and timing. In stage 3-RS (Require Side), animals were required to initiate a trial by poking in the center port, and to respond to the stimulus by poking in the reward ports. There was no delay between the center poke and the stimulus presentation. Water was only delivered after the animal poked in the appropriate reward port for that trial, but poking the other ports did not finish the trial. If animals had not collected reward after a period of 4 s, the trial finished and the animal had to initiate a new trial. Animals advanced to the next stage after performing more than 200 trials in one session. In stage 4-ID (Increase Delay), the delay between the center poke and the onset of the stimulus was increased. Initially, animals were allowed to withdraw from the center port at any time after the stimulus onset. The delay was first increased in increments of 10 ms every 10 valid trials. After 50 trials it was increased to 100 ± 20 ms, and at 100 trials to 200 ± 50 ms. After 150 trials animals were required to stay in the center port until the offset of the stimulus. An early withdrawal resulted in the termination of the trial. Animals advanced to the next stage after performing more than 200 trials in one session, or 3 consecutive sessions each with more than 100 trials. At this point, the delay to the stimulus was increased to its final value of 300 ± 50 ms.

Stage 5-RC (Required Correct) consisted of the full frequency discrimination task (3 vs. 16.3 kHz). In this stage, reward was delivered only if the animal chose the correct reward port after leaving the center port. Error trials resulted in a timeout of 4 s during which all ports were inactive. If animals showed a bias toward one reward port, we activated a correction procedure for the following session. The method consisted of repeating the previous trial if incorrect. This correction procedure method was not activated beyond this stage. Animals advanced to the next stage after two consecutive sessions with 80% correct trials for each stimulus type.

In the next stage, 6-SB (Switch in Blocks), each session started in one of the two possible contingencies, either 3 vs. 7 kHz, or 7 vs. 16.3 kHz. Contingencies alternated every 300 valid trials. The initial contingency was also alternated from one day to the next. Previous studies from our group showed that performance for reversing stimuli (in this case, 7 kHz) is lower than for non-reversing stimuli (3 and 16.3 kHz) (Jaramillo et al., 2014). We therefore set a lower criterion for advancing to the next stage compared to the previous transition. Animals advanced to the next stage after one session with 70% correct trials for the reversing stimuli under each of the two contingencies.

The last stage (7-OK) was achieved once animals were able to successfully switch between contingencies within a single session, and was used to estimate psychometric performance along a range of stimulus frequencies. In this stage, 10% of the trials contained sounds with center frequency logarithmically spaced between 3 and 16.3 kHz. The correct reward port for each stimulus was defined according to boundaries located at the geometric mean between the 3 frequencies used for training: 4.5 kHz for one contingency, 10.6 kHz for the other. The fraction of stimuli of each frequency was balanced to obtain the same number of left- and right-reward trials.

### 2.5. Analysis of behavioral performance

Data were analyzed using in-house software developed in *Python* (www.python.org). Performance traces for a single session (**Figures 5A,B**) were calculated by averaging over a moving window (40 trials long) the fraction of correct trials for each stimulus. To calculate the number of trials needed to switch between categorization contingencies (**Figure 5**) we fit an exponential function to the performance in response to the reversing stimulus after a contingency switch. We first estimated the fraction of correct choices for each trial after the switch, by averaging across 20 sessions of stage 7-OK. No time-averaging was applied (the moving window was 1 trial long). We pooled together switches from low-to-high and high-to-low boundary contingencies, and fit the function:

(1) $$f(k)=A\left(1-e^{-k/\tau }\right)+I$$

where *k* is the trial index after a switch, and 1 − *I* is the performance at the end of the previous block, estimated from the last 100 trials before a switch. The parameters *A* and τ are related to the asymptotic performance and the speed of change in performance respectively. The number of trials to switch was calculated as the point at which the exponential fit crossed the 50% chance level performance.

Psychometric curves were fit using the Python module *Psignifit 3.0* (Fründ et al., 2011). Briefly, a constrained maximum likelihood method was used to fit a logistic function with 4 parameters: α (the 50% threshold, or boundary), 1/β (the slope of the curve), γ (the lower asymptote), and λ (the higher asymptote).

(2) $$\begin{array}{l} \Psi (x)=\gamma +(1-\gamma -\lambda )\frac{1}{1+\exp\;(-g(x))}\\ g(x)=\frac{x-\alpha }{\beta } \end{array}$$

## 3. Results

To compare performance, speed of learning, and adaptability between mice and rats, we used a sound categorization task in which the category definitions changed within a single behavioral session (Figure 1). Animals had to report if a target sound (100 ms long) was of high or low frequency by poking in either the left or right reward port after trial initiation. The boundary that defined what sounds belonged to the low- or high-frequency categories varied between two possible values every 300 trials.

We first evaluated how fast 10 individuals of each species advanced through the different stages of training (Table 2), and then compared the final performance across the two cohorts. Animals were trained in 1 h sessions every week-day and the amount of reward was scaled according to the normal consumption for each species (Table 1). Training parameters (session duration, reward amount, etc.) were set to make conditions as similar as possible for all individuals and across the two species, instead of trying to minimize the training time or maximize performance for each individual.

### 3.1. Rats learned the task faster than mice

**Figure 2:**
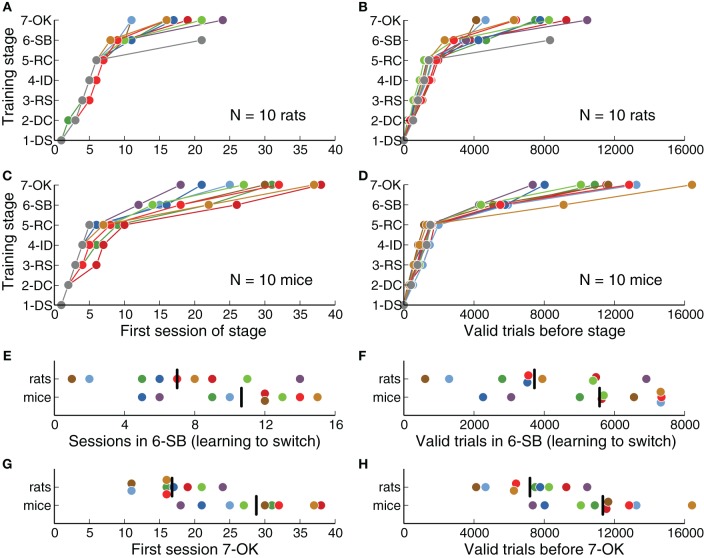
**Rats learned the task faster than mice**. **(A)** Training stage as a function of number of sessions for each rat. One rat never reached the last stage. **(B)** Training stage as a function of the number of valid trials (those in which the animal waited long enough for the stimulus). **(C)** Same as (**A)** for each mouse. One mouse never reached stage 6-SB. **(D)** Same as **(B)** for each mouse. **(E)** Number of sessions needed to learn to switch between contingencies (stage 6-SB), after the basic discrimination was learned (stage 5-RC). **(F)** Number of trials needed to learn to switch, after the basic discrimination was learned. **(G)** Overall number of sessions to reach the last stage of training. **(H)** Overall number of valid trials to reach the last stage of training. Black vertical lines indicate the average for each species.

We defined a fixed criterion to advance from one training stage to the next (Table 2), and animals were required to perform at least one full session on each stage. We quantified how many sessions were needed on each stage for animals to successfully learn the task (Figures 2A,C). We found that rats learned the basic discrimination task (reaching stage 6-SB, Switch in Blocks) faster than mice (*p* = 0.0014, rank-sum test). On average, rats reached the criterion of 80% correct for all stimuli by session 9.8 ± 0.9 (mean ± *SD*), while mice took 18.1 ± 4.2 sessions. From all stages, stage 5-RC (Required Correct), in which animals learned to discriminate sounds, showed the largest difference between mice and rats (7 sessions on average, *p* = 0.0003, rank-sum test). This stage was responsible for most of the difference in the overall length of training. One mouse never learned the discrimination task, although this animal showed no impairment in initiating trials or collecting reward. One rat never reached the final stage (7-OK). These animals were excluded from subsequent analysis.

In stage 6-SB (Switch in Blocks), animals learned to switch between two categorization contingencies within a single behavioral session. The average number of sessions spent learning to switch (after learning to discriminate sounds) was less for rats than for mice (Figure 2E), although this difference was not statistically significant (*p* = 0.07, rank-sum test). Overall, rats reached the final stage (7-OK) faster than mice (Figure 2G) (*p* = 0.0014, rank-sum test), although the distributions largely overlapped and some mice were as fast as the average rat.

We also quantified the number of trials needed on each stage (Figures 2B,D) and found results consistent with our analysis of the number of sessions. Stage 5-RC (Required Correct), showed the largest difference between species (rats required 2320 fewer trials on average, *p* = 0.0013, rank-sum test). Moreover, rats achieved the final stage in fewer trials than mice (Figure 2H, *p* = 0.0054, rank-sum test), although the number of trials spent learning to switch was not significantly different (Figure 2F, *p* = 0.057, rank-sum test). There was no apparent correlation between the trials required to complete stages 5-RC (Required Correct) and 6-SB (Switch in Blocks) for either mice or rats (|ρ| < 0.12, *p* = 0.57, Spearman correlation).

### 3.2. Rats and mice achieved similar performance levels

**Figure 3:**
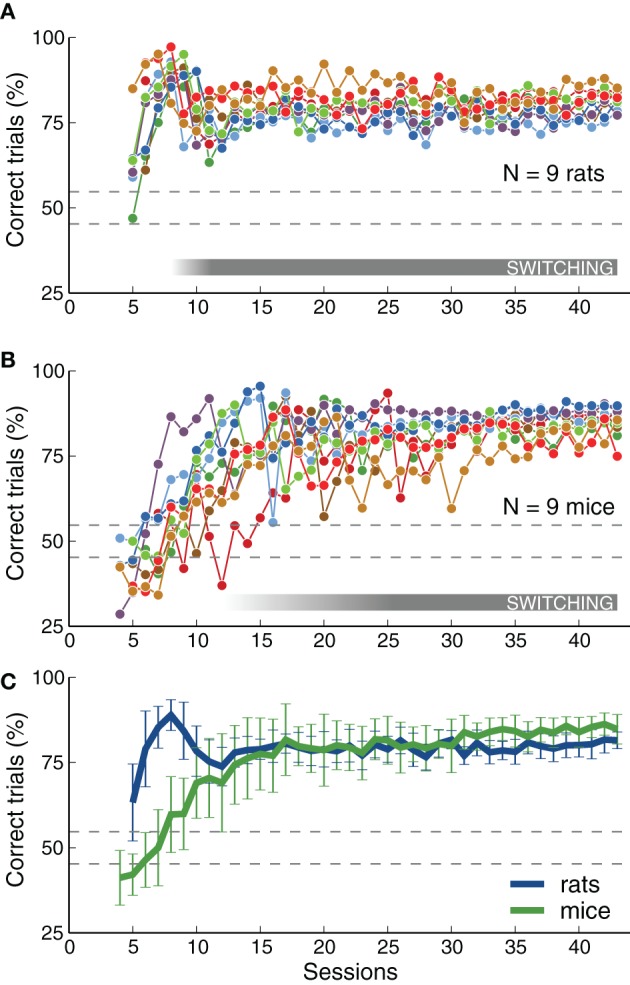
**Rats and mice achieved similar levels of performance**. **(A)** Percentage of correct trials (ignoring early withdrawals) as a function of number of sessions. Dotted lines indicate chance level range (assuming 500 trials, *p* = 0.01). The gray bar indicates when animals were required to switch between contingencies (not all animals reached that stage at the same time). The plot includes only sessions from stage 5-RC or after (the first stage that required correct responses on the first attempt). Some rats start this stage with performance above chance. The rat that did not reach the final stage is not included. **(B)** Same as **(A)** for mice. The mouse that did not reach the final stage is not included. Some mice had bias-correction activated and show performance below chance. **(C)** Average performance across animals of each species. Mice achieved performance level as high as rats, but after more training sessions. Error bars correspond to standard deviations. This figure includes all trials (easy and difficult) in each session. Final performance computed for the easiest frequencies alone is higher than that plotted here: above 80% for all animals, and above 90% for most.

We quantified changes in performance levels as animals learned the task. Figure 3 shows the overall performance for each training session for each animal across several stages. The plots illustrate how, as described in the previous section, rats achieved high performance levels faster than mice on average. Although both species achieved a similar fraction of correct trials per session (Figure 3C), mice showed a slightly higher average performance on the last stages (*p* < 0.05 rank-sum test, for several consecutive sessions after reaching stage 6-SB, Switch in Blocks). This observation will be discussed in more detail in the next section when comparing psychometric slopes between the two species. Each point in the plot represents the average for each entire session, including transition trials between blocks, and trials with intermediate frequencies on psychometric sessions. Performance for the easiest frequencies (3 and 16.3 kHz) was, as expected, higher than these values: above 80% for all animals, and above 90% for most.

As expected, there was a decay in performance at the transition between the frequency discrimination stage (5-RC, Required Correct) and the contingency switching stage (6-SB, Switch in Blocks), illustrated with a gray horizontal bar in Figures 3A,B. This is visible in the average curve for rats around session 9 (in Figure 3C). Mice showed a similar change in performance at this transition, although this feature in smoothed out in the average plot because the transition session was highly variable across mice. Figure 3 only includes sessions after animals were required to make the correct choice immediately after stimulus presentation (stage 5-RC and after). Some rats had already learn to associate sounds with the appropriate reward port and show performance above chance level at the beginning of the plot. In contrast, mice were either at chance level or had the bias-correction mode activated, resulting in performances below chance. Note that for an average of 500 trials per session, performance below 56% would be considered at chance (*p* < 0.01, binomial test), as indicated by the dotted lines in Figure 3.

**Figure 4:**
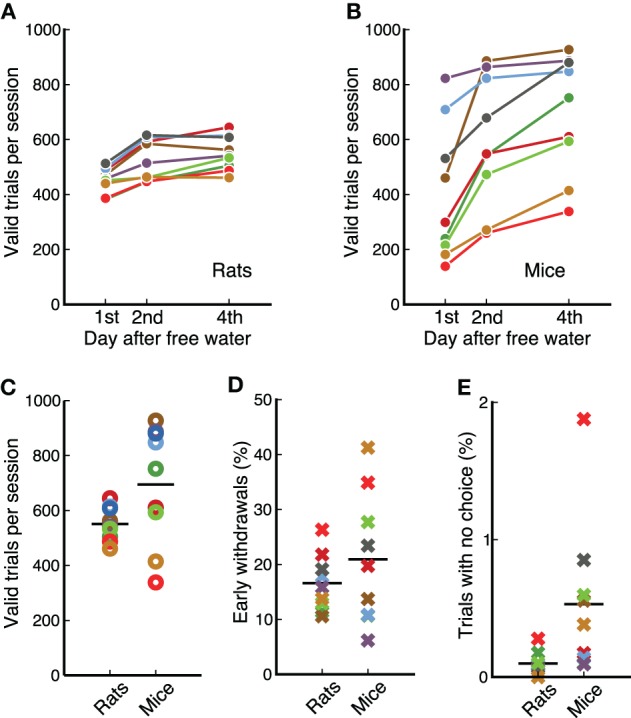
**Rats and mice performed a similar number of trials per session**. **(A)** Average valid trials per session for each rat on different days after having free water. Animals performed fewer trials on the first day. Each dot is the average across 4 days from different weeks. **(B)** Same as **(A)** for mice. **(C)** Comparison between mice and rats (data from day 4 after free water). Average number of trials is similar between species, but variability is higher across mice than across rats. **(D)** Percentage of trials in which each animal incorrectly withdrew from the center port before the offset of the sound. Data from the first 4 sessions of stage 7-OK for each animal. **(E)** Percentage of trials in which each animal chose no reward port after the target was presented. Data from the first 4 sessions of stage 7-OK for each animal.

Physiological studies of the mechanisms underlying complex behaviors often require hundreds of trial in a single experimental session. Our measurements show that animals perform several hundred valid trials per session (Figure 4), enabling a quantification of changes in the response properties of neurons as animals switch between contingencies. Animals received free water on weekends resulting in a clear difference between the number of trials on the first day of the week and subsequent days for both species (*p* = 0.0077, for rats and for mice, Wilcoxon signed-rank test). This effect was less pronounced after the first day: from first to second day rats increased the number of trials by 72 ± 37; from second to fourth day only by 25 ± 31 (Figure 4A). Similarly for mice, the first increase was 194 ± 116, while the second was 101 ± 68 (Figure 4B).

We found no statistically reliable difference in the average number of trials per session between mice and rats for the reward amounts used in our study (*p* = 0.12, rank-sum test), although the variability across animals was larger in the cohort of mice (Figure 4C). Previous measurements in our laboratory have shown that rats can perform more trials than those presented here if behavioral sessions are longer than 1 h.

Animals were required to stay in the center port during an initial silent delay (250–250 ms) plus the whole duration of the target sound (100 ms). Figure 4D shows the fraction of trials in which animals left the center port before the offset of the sound, for the first 4 session of the final stage, 7-OK. Early withdrawals were about 20% on average for both mice and rats (*p* = 0.56, rank-sum test), although variability was higher for mice. Analysis of subsequent sessions showed that the average for rats remained in the range 15–20%, while the average for mice fluctuated in the range 20–30%. The fraction of trials in which animals listened to the whole duration of the target sound but did not make a choice was less than 1% for all animals except one mouse (Figure 4E). The average was slightly lower for rats (*p* = 0.007, rank-sum test). Averages remained under 1% on subsequent sessions.

### 3.3. Rats and mice switched equally fast between categorization contingencies

**Figure 5:**
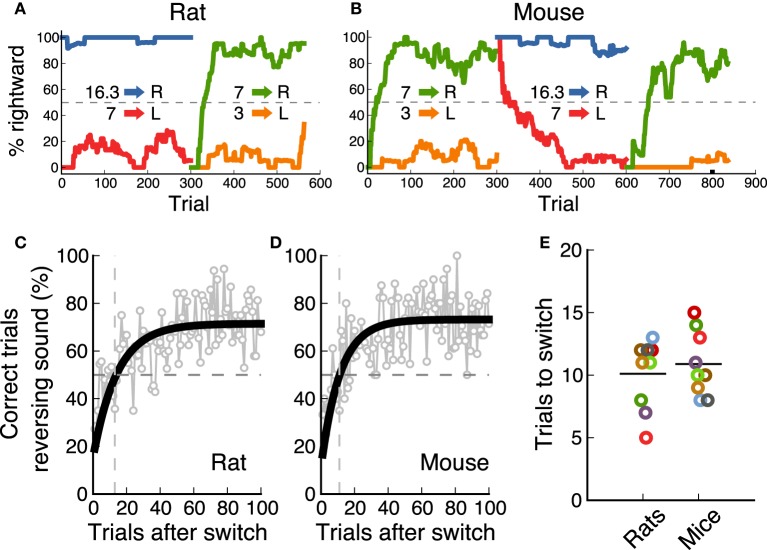
**Rats and mice switched equally fast between contingencies**. **(A)** Example of the performance of a rat (one session) as the contingency changes. **(B)** Example session for a mouse. **(C)** Performance of one rat after the contingency switch. In gray is the average for each trial across several sessions. In black is an exponential fit. The dotted line indicates when performance crosses chance level. **(D)** same as **(C)** for one mouse. **(E)** Comparison of speed of switching for rats and mice. Each dot corresponds to the number of trials it took each animal to cross chance level after a switch.

In the final task, the category boundary switched between two possible values from one block of 300 trials to the next. As a result, animals were required to associate a different reward port to the middle frequency sound (7 kHz) on each block. Nine out of ten animals from each species achieved performance levels above 70% for this reversing stimulus on both contingencies (Figure 2). Performance for the non-reversing stimuli (3 and 16.3 kHz) was always above this level. Figures 5A,B show an example session from one rat and one mouse respectively, as they switch between contingencies (performance for each sound-action association is shown in a different color). These examples show how animals rapidly modified their response to the 7 kHz after a contingency change. We estimated the speed of switching to a new sound-action association by averaging performance for each trial from several sessions, and calculating when an exponential fit crossed the 50% chance performance level (Figures 5C,D). We found that both rats and mice needed about 10 trials to switch between contingencies (Figure 5E, *p* = 0.72, rank-sum test).

**Figure 6:**
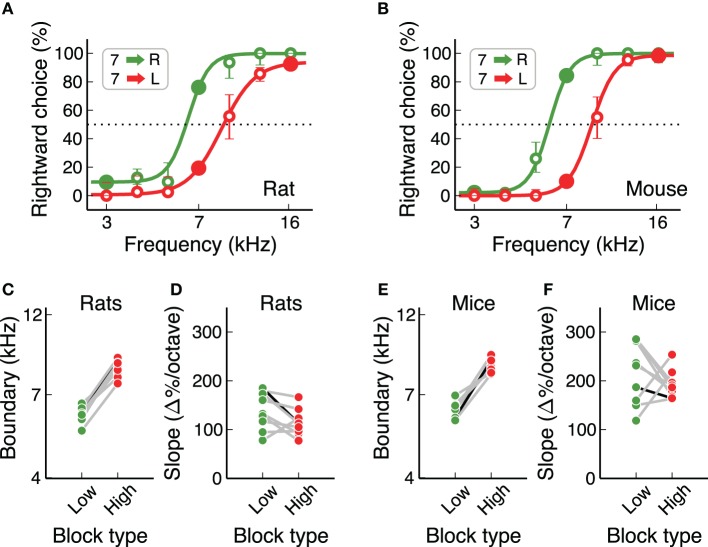
**Rats and mice had similar subjective categorization boundaries**. **(A)** Example psychometric curves estimated for one rat on each contingency. Data pooled from several sessions. Error bars are 95% confidence intervals. **(B)** Same a **(A)** for one mouse. **(C,D)** Subjective categorization boundary and slope of the psychometric curve for all rats. The black line corresponds to the example animal in **(A)**. **(E,F)** Same as **(C,D)** for mice. Slopes were higher for mice, but subjective boundaries were the same for both species.

We also estimated the subjective categorization boundary from each individual under each contingency (Figure 6). We first quantified performance in response to several sound frequencies in addition to the three stimuli used for training, interleaved throughout the session. We then fitted a logistic function to the psychometric performance and evaluated two parameters: the subjective category boundary and the slope of the curve. Both sets of animals showed a clear change in subjective category boundary between the two contingencies (Figures 6C,E, *p* = 0.0077 for both rats and mice, Wilcoxon sign-rank test), but no consistent change in slope (*p* = 0.07 for rats, *p* = 0.37 for mice, Wilcoxon sign-rank test). The change in subjective category boundary as animals switched from one contingency to the other was not different between mice and rats (*p* = 0.89 rank-sum test), but mice showed slightly steeper slopes than rats (*p* = 0.019 on the low-boundary block, *p* < 0.001 on the high-boundary block).

### 3.4. C57BL/6J mice can perform the task after several hundred days

**Figure 7:**
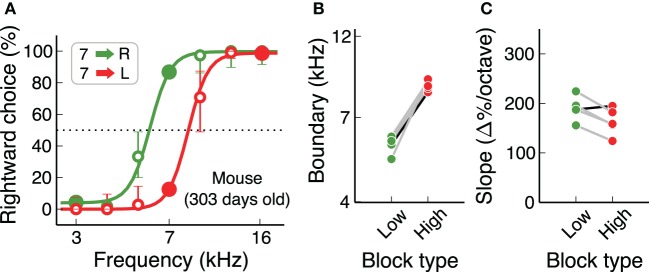
**Mice continue to perform the task for many months**. **(A)** Example psychometric curve for a mouse older than 300 days, showing that any age-induced deafness in the C57Bl/6J strain has not produced any visible effect at this age. **(B,C)** Subjective categorization boundary and slope of the psychometric curve for 5 mice older than 300 days. The black line corresponds to the example animal in **(A)**. Values are similar to those from younger mice.

An important consideration when working with mice is that some strains suffer from early loss of hearing sensitivity (Zheng et al., 1999). C57Bl/6J, a common background for transgenics used in the study of neural circuits, show elevated auditory brainstem response thresholds at 700 days of age (60 dB higher than normal), but not at 200 days of age. For the study of auditory behaviors, it is therefore necessary to test if hearing loss affects performance in the time-scale of the experiments. We found that C57Bl/6J mice can perform the auditory flexible categorization task for several hundred days without major impairments in performance. Figure 7 shows psychometric curves for each contingency for a 300-day old mouse, as well as categorization boundaries and psychometric slopes of 4 more animals of similar age. These results are comparable to those from our initial cohort of mice (Figures 6E,F).

## 4. Discussion

Our study quantified the performance of mice and rats in an adaptive decision-making task. The flexible sound categorization task used here required animals to switch between two possible interpretations of a stimulus in order to successfully collect reward. We compared three main behavioral features between the two species: (1) how long it took animals to learn the task, (2) how well they adapted between categorization contingencies, and (3) how fast they adapted between contingencies. We found that under our training conditions: (1) rats were faster than mice at learning the task, although the distributions overlapped; (2) both mice and rats successfully changed their subjective categorization boundaries after changes in contingencies; and (3) both species adapted equally fast between contingencies. Although both cohorts performed the task well, measurements after the last stage of training showed slightly higher overall performance levels and steeper psychometric slopes for mice than for rats.

In this study, we set the duration of behavioral sessions to 1 h for all stages of training, kept the amount of reward per trial constant, and used the same criteria to advanced each individual from one stage to the next. Consistency in training parameters helped us compare directly the two species, the main goal of the study. Other objectives, for example minimizing training times for each animal, may require adjusting parameters for each individual.

### 4.1. Rodents in the study of the neural basis of adaptive behavior

There are opposing constraints in the choice of a model organism for the study of the neural basis of complex behaviors: researchers need animals that allow for a high degree of experimentation, and yet be capable of performing complex cognitive tasks. Studies of the physiological basis of decision-making have traditionally been carried out on non-human primates (Parker and Newsome, 1998; Gold and Shadlen, 2007), motivated by the similarity of their brain anatomy to that of humans. Smaller mammals, rodents in particular, provide several advantages over primates for physiological experiments: faster breeding times, lower costs, smaller housing needs, and currently, easier access to molecular and genetic tools. And although rodents may not have the cognitive abilities of primates, they can perform a variety of adaptive decision-making tasks (Dudchenko, 2004; Jaramillo and Zador, 2011; Bissonette and Powell, 2012; Izquierdo and Belcher, 2012; Jaramillo et al., 2014).

Rodents have been used for many decades in studies of learning and sensory perception. However, several of the behavioral paradigms employed required subject handling between trials (affecting among other things, the number of trials achievable per session), or measurements that relied on the precision of a human observer (when estimating for example freezing times). Several strategies have been implemented to minimize these issues. In particular, automatic systems have been developed allowing animals to initiate trials without intervention of the experimenter and enabling automatic scoring (Wahlsten et al., 2003; Schaefer and Claridge-Chang, 2012). These systems permit the collection of hundreds of trials per session, a feature often required in physiological experiments. In addition, the use of quantitative behavioral assays allow for detailed characterization of performance and, in turn, a deeper investigation of potential mechanisms underlying decision-making. These advances enable performing in rodents similar psychophysical measurements to those classically studied in humans. The paradigm described in this manuscript is an example of such quantitative characterization of behavior.

Animals could use various strategies for solving the task described in this study. First, animals may evaluate the outcome of responses to the reversing frequency (7 kHz) to update their sound-action associations. Alternatively, the appearance of an extreme stimulus (3 or 16.3 kHz) could be used as a cue indicating the most likely contingency. These strategies are non-exclusive, and animals may be taking advantage of both. Independent of the strategy used, however, animals consistently change the action associated with a subset of stimuli (in particular, 7 kHz), and the observation that animals generalize to other frequencies is well captured by a model in which an internal category boundary shifts between blocks of trials (Jaramillo et al., 2014). Similarly, there are at least two possible mechanisms for this adaptation in behavior: (1) changes in synaptic strength in the sensory-motor pathway that result in different behavioral responses for the same sound; or (2) integration of sensory and context information without long-term synaptic changes in the sensory-motor pathway. The former correspond to mechanisms hypothesized for learning and memory (Martin et al., 2000), the latter to mechanisms of selective attention (Jaramillo and Pearlmutter, 2007), both defining features of cognition. Although behavioral measurements alone may not dissociate between these possibilities, techniques for physiological analysis of neural circuits in the mouse have the potential to reveal the mechanisms that mediate this rapid adaptation in the interpretation of sounds.

### 4.2. Mice vs. rats

The rat has been the preferred rodent model in the study of the neurophysiological basis of behavior (Buzsàki et al., 1989; Dudchenko, 2004; Izquierdo and Belcher, 2012). The mouse, in contrast, has been favored when genetic manipulations are required (Crawley, 2008). When choosing between rat and mouse for investigating the neural basis of complex behaviors one needs to take into account several factors. Because of their size, rats can accommodate larger chronically implanted devices than mice. It is also easier to target specific regions in animals with larger brains. Mice, being smaller, are less expensive to house and less time consuming (e.g., during tissue processing). Their size also makes mice better suited for experiments that require covering a larger extent of brain tissue, for example when delivering light for optogenetic experiments or trying to reach deeper cortical layers with two-photon imaging. But the main experimental advantage of the mouse over the rat is the larger number of available transgenic lines that enable cell-type specificity in neurophysiological experiments (Huang and Zeng, 2013). Although, there are now techniques for manipulating the genome of both species (Filipiak and Saunders, 2006; Witten et al., 2011), the mouse has had a long head start as illustrated by the thousands of transgenic lines available from The Jackson Laboratory repository.

Despite these advantages of mouse over rat, it is still debated whether the mouse can perform the type of adaptive decision-making tasks that have been successful in studies with rats (Carandini and Churchland, 2013). Most comparisons of behavioral performance between mice and rats, including our study, have used tasks originally devised for rats. This potential confound may explain why rats have been found to be better in a variety of tasks (Whishaw and Tomie, 1996). Behavioral paradigms for mice should therefore be designed taking into account the ethological validity of the task requirements. Small changes in the training apparatus and protocols can make a difference. For instance, previous measurements in our lab indicated that rats may not need the first stage of training (1-DS) presented in this study, but mice benefit greatly from this conditioning stage.

Mice and rats have been directly compared during memory-guided navigation tasks (Whishaw and Tomie, 1996; Frick et al., 2000; Cressant et al., 2007) and tasks that require sensory-driven decisions (Prusky et al., 2000; Mayrhofer et al., 2013). Behavior of the two species was similar in the navigation tasks when evaluated in the dry-land maze. Differences found in water-maze tasks seem to be accounted for mostly by differences in swimming rather than memory capabilities. Nevertheless, the two species seem to use different strategies for solving some of these tasks, and whenever differences in performance levels were found, results favored the rat. Similar conclusions have been reached for sensory-driven decision tasks. In a two-alternative forced choice paradigm for vibrotactile discrimination, the performance of mice and rats was found to be very similar when quantifying psychometric curves, reaction times, learning rates, and trial omissions (Mayrhofer et al., 2013). In a visual discrimination task, both species learned the task well, although rats showed higher visual acuity and (at least in the examples reported) learned the task faster (Prusky et al., 2000).

In addition to these direct comparisons, researchers have evaluated behavioral performance of mice during tasks originally devised for rats. These studies have shown that mice are capable of performing tasks requiring delayed matching-to-position (Goto et al., 2010), visuospatial attention (Humby et al., 1999), attentional shifts (Bissonette et al., 2008), or visual reversals (Izquierdo et al., 2006). Importantly, researchers are now using tasks that make possible detailed psychometric measurements from mice (Prusky et al., 2000; O'Connor et al., 2010; Busse et al., 2011; Tai et al., 2012; Glickfeld et al., 2013).

The mouse is uniquely placed at the interface between experimental access and behavioral complexity, making it an ideal model for the study of adaptive decision-making. Successful behavioral paradigms, however, rely on targeting designs to the idiosyncrasies of the mouse from the outset, rather than simply assuming that mice are little rats.

## Author contributions

Santiago Jaramillo and Anthony M. Zador designed the study. Santiago Jaramillo collected and analyzed the data. Santiago Jaramillo and Anthony M. Zador wrote the paper.

## Funding

This work was supported by the National Institutes of Health (Grants R01DC012565 and R01NS088649) and the Swartz Foundation.

### Conflict of interest statement

The authors declare that the research was conducted in the absence of any commercial or financial relationships that could be construed as a potential conflict of interest.
